# Inhibition of Bruton's Tyrosine Kinase Protects Against Burn Sepsis-Induced Intestinal Injury

**DOI:** 10.3389/fmed.2022.809289

**Published:** 2022-02-24

**Authors:** Jia Wan, Xi Yu, Jia-Qi Niu, Le Qiu, Fei Wang, Xu-Lin Chen

**Affiliations:** Department of Burns, The First Affiliated Hospital of Anhui Medical University, Hefei, China

**Keywords:** Bruton's tyrosine kinase, intestinal injury, burns, sepsis, oxidative stress

## Abstract

This study aimed to investigate the role and molecular mechanisms of Bruton's tyrosine kinase (BTK), a member of the Tec family in burn sepsis-induced intestinal injury. Eighty C57BL/6 mice were randomly divided into four groups: the sham group, the burn group, the burn + sepsis group, and the burn + sepsis + LFM-A13 (a selective BTK inhibitor) group. The dynamic expression profiles of BTK and p-BTK in the intestine were measured by Western blot analysis. Intestinal histopathological changes and cellular apoptosis were determined. Inflammatory cytokines in serum and intestinal tissue were examined through enzyme-linked immunosorbent assay. Myeloperoxidase (MPO) activity was determined *via* a colorimetric assay. Intestinal p-BTK expression in the burn+sepsis group was significantly increased compared with that in the sham and burn groups. In the burn + sepsis group, the p-BTK expression level increased over time, peaked at 12, and then decreased at 24 h. LFM-A13 administration significantly inhibited p-BTK expression in the intestine. In contrast to the sham and burn groups, the burn + sepsis group exhibited obvious histopathological changes, which gradually aggravated over time. LFM-A13 also reduced the histopathological changes and cellular apoptosis in intestinal tissues, inhibited the inflammatory cytokines IL-4, IL-6, and TNF-α in serum and intestinal tissues, and significantly inhibited the increase in intestinal MPO activity induced by burn sepsis. BTK activation is one important aspect of the signaling event that may mediate the release of the anti-inflammatory cytokine IL-4 and the pro-inflammatory cytokines IL-6 and TNF-α; oxidative stress; and intestinal cell apoptosis. Thus, it contributes to burn sepsis-induced intestinal injury.

## Introduction

Burn injuries, as one of the common clinical critical illnesses worldwide caused by thermal aggressions, have caused high morbidity and mortality rates, especially in low and middle-income countries (1). Burns affect the integrity of the skin, which acts as the protective organ that prevents the body from experiencing microbial invasion; extensive burn injuries are highly prone to inflicting deep damages, such as severe infections, complications, and sepsis, to the body (2). Sepsis is a multifactorial and complex pathophysiological syndrome caused by systemic infection with exaggerated inflammatory response and leads to severe consequences, such as shock, multiple organ dysfunction, and even death (3). According to the recent statistics of the United States, the mortality rate among patients with sepsis is approximately 20–50%, making sepsis a major health and economic concern (4, 5). A series of diagnostic and treatment measures, including appropriate antimicrobial treatment, infection control measures, fluid and nutritional support, debridement, and wound closure, have been gradually improved in recent years, considerably enhancing the prevention and treatment of burn sepsis and improving the taking rate (6, 7). However, the pathogenesis and knowledge of sepsis remain insufficient even after decades of exploration by researchers, resulting in the lack of theoretical guidance for the further treatment of this disease.

As generally known, the main functions of the intestinal tract are digestion and absorption to provide essential energy to organisms, and the intestinal tract is often considered to play a passive role in the development of sepsis and the actions of the body against pathogenic microorganisms (8). However, several scholars have found that during the early stage of sepsis, some abnormal phenomena occur during intestinal metabolism, that is, the intestinal tract is considered to be the “engine” in the process of sepsis-induced multiorgan dysfunction syndrome (MODS) (9). Therefore, exploring the relationship between the dysfunction of the intestinal mucosal barrier and the occurrence of sepsis is essential (10). With the deepening of research in recent years, researchers have discovered that the dysfunction of the intestinal mucosal barrier leads to the imbalance of intestinal microbiota; the entry of endotoxins into the blood through the barrier may induce the release of large amounts of inflammatory factors and thus accelerate the development of sepsis (11). In addition, intestinal epithelial and endothelial cells are further disrupted when sepsis-induced excessive inflammatory responses occur, further exacerbating intestinal permeability and intestinal flora imbalance and thus inducing a vicious cycle of sepsis (12). Moreover, these interactions lead to the activation of large numbers of genes, inflammatory cytokines (e.g., the proinflammatory cytokines TNF-α andIL-6 and the anti-inflammatory cytokine IL-4), and chemokines that are released by macrophages under the mediation of downstream signaling molecules involved in inflammatory responses (13). These processes enhance the systemic inflammatory response and further aggravate damage to the viscera. Therefore, the treatment of sepsis by blocking these signaling molecules is considered as a promising approach.

Recent studies have shown that Gram-negative bacteria are one of the prominent pathogens that cause sepsis, and the key molecule that can induce sepsis is the lipopolysaccharide (LPS) present on their surfaces (14, 15). As a Toll-like receptor (TLR), an important protein molecule involved in non-specific immunity, the TLR4 molecule could bind with the antigen molecule LPS, activate the signaling pathways of TRIF and MyD88, and then stimulate their downstream signaling pathways, this causing systemic or local inflammatory reactions in the body (16, 17). Therefore, TLR4 is a promising target in the treatment and intervention of sepsis. However, blocking the whole TLR4 pathway may result in the failure of follow-up anti-infective treatment given that burn sepsis is an aggravation of systemic inflammatory response.

The Tec family, which has been widely studied in recent years, is a series of cytoplasmic tyrosine protein kinase molecules and non-receptor tyrosine kinases (TKs) that are mainly expressed in lymphocytes and myeloid cells (18). BTK, a member of the Tec family, is involved in the growth and function of B cells; it is closely associated with a variety of B-cell lymphoid tissue disorders and is also a key kinase in the B-cell antigen receptor signaling pathway (19). In addition, in human mono-nuclear cell lines and primary human mono-nuclear cells, BTK is one of the important signaling molecules in the TLR signal pathway and is phosphorylated after LPS stimulation and interacts with multiple components of the TLR pathway (20). Therefore, the selective blocking of BTK may provide greater benefits to patients than blocking the whole TLR4 pathway, and BTK may be an ideal target for intervention in patients under the condition of systemic inflammatory response and organ dysfunction caused by burn sepsis. LFM-A13, the first inhibitor of BTK, was used as the selective blocking of BTK in this study (21).

Therefore, the present study was designed to determine the effects of LFM-A13 on burn-sepsis-induced intestinal injury; the intestinal activation of BTK; the expression changes of the anti-inflammatory cytokine IL-4 and the proinflammatory cytokines IL-6 and TNF-α; oxidative stress; and intestinal cell apoptosis in the intestinal injury of mice induced by burn sepsis.

## Materials and Methods

### Animals and Reagents

Male C57BL/6 mice with average weights of 23–26 g were purchased from the Corporation of Lingchang Biological Technology (Shanghai, China). The animals were allowed to acclimate to their surroundings for 1 week. LPS was purchased from Sigma (USA). LFM-A13 was purchased from Topscience (Shanghai, China). Kits for protein extraction, BCA protein assay, SDS-PAGE gel preparation, Western blot analysis, hematoxylin and eosin (H&E staining), TUNEL apoptosis assay, mouse IL-4 enzyme-linked immunosorbent assay (ELISA), mouse IL-6 ELISA, mouse TNF-α ELISA, and myeloperoxidase (MPO) colorimetric activity assay were purchased from KeyGEN (Nanjing, China). Neutral gum, methanol, ethanol, and xylene were purchased from Sinopharm Chemical Reagent Co., Ltd (Shanghai, China). Triton-X100 was purchased from KeyGEN (Nanjing, China). Rabbit anti-GAPDH (10B8) and goat antirabbit IgG-HRP were purchased from KeyGEN (Nanjing, China). Rabbit anti-p-BTK (ab52192) was purchased from Abcam (Shanghai, China). Rabbit anti-BTK (DF6472) was purchased from Affinity Biosciences (Cincinnati, USA).

### Animal Model Preparation and Groups

This study was performed in accordance with the Guide for the Care and Use of Laboratory Animals of the National Institutes of Health. The protocol was approved by the Anhui Medical University Ethics Committee of Animal Experiments in Hefei city, China (Permit Number: 20160188).

Mice were anesthetized intraperitoneally with pentobarbital sodium (30 mg/kg), and their back skin were shaved. Eighty animals were randomly divided into four groups: sham group (*n* = 8), burn group (*n* = 8), burn + sepsis group (*n* = 32), and burn + sepsis + LFM-A13 group (*n* = 32). Animals in the sham group were sacrificed after anesthesia. The mice received full-thickness scald burns on 10% of their total body surface area on their dorsal area by immersion for 15 s in a 100°C water bath. Animals in the burn group were resuscitated immediately with the intraperitoneal administration of normal saline (10 mg/kg). Animals in the burn + sepsis group were injected with LPS (10 mg/kg) intraperitoneally after scalding to establish the sepsis model. Animals in the burn + sepsis + LFM-A13 group received intervention through the intraperitoneal administration of LFM-A13 immediately after scalding before LPS injection. According to our previous study, the dose of LFM-A13 was set at 10 mg/kg (22). Animals in the burn + sepsis and burn + sepsis + LFM-A13 groups were sacrificed at 0, 8, 12, and 24 h (8 animals per time-point) after dosing.

### Sample Collection

A portion of intestinal tissue was removed from each mouse and shock frozen in liquid nitrogen for Western blot analysis. Another portion of intestinal tissue was collected for histopathological examination (H&E staining), cellular apoptosis (TUNEL assay), inflammatory cytokine detection (ELISA), and MPO activity determination (MPO colorimetric activity assay).

### Measurement of Total BTK and p-BTK Protein Levels *via* Western Blot Analysis

Total proteins were extracted from intestinal tissue samples for Western blot analysis. The concentration of protein was determined by using the Bradford method established by Bradford in 1976. The protein samples were separated through SDS-PAGE, transferred to nitrocellulose membranes, and oscillated in 5% blocking solution at room temperature. The membranes were incubated with primary antibodies on a shaker at 4°C overnight. The membranes were washed three times with TBST, incubated with horseradish peroxidase-conjugated secondary antibodies for 1 h, and washed three times with TBST for color development. Blots were imaged by using a G:BOX ChemiXR5 imaging system, and the gray values were analyzed by using Gel-pro 32 analysis software.

### Histopathological Examination *via* H&E Staining

Intestinal tissues were fixed, dehydrated, and embedded in paraffin and sectioned to a thickness of 5 μm for H&E staining. The sections were observed through optical microscopy (×400) with three fields of vision that were randomly selected from each section; photographed; and scored for histopathological changes. The scoring criteria were as follows: 0 represents neatly arranged and complete glandular architecture, columnar-lined epithelia, and abundant goblet cells, and each one added point represents glandular atrophy, epithelial thinning, and absent or shedding goblet cells. Gland absence is represented by a score of three points.

### Measurement of Cellular Apoptosis *via* TUNEL Assay

Cellular apoptosis was detected through TUNEL assay by using a TUNEL apoptosis assay kit. Paraffin-embedded sections were dewaxed, dehydrated, and rehydrated for the TUNEL assay. Slides containing cells were treated with 0.1% Triton X-100 for 15 min at room temperature. Next, each sample was incubated with 10 μl of proteinase K solution and 90 μl of PBS for 5 min and washed with PBS. Each sample was incubated with 50 μl of the TUNEL reaction mixture for 1 h at 37°C and washed with PBS. DAB solution was utilized for color development. The slides were observed by using an optical microscope, and the apoptotic index (AI) was calculated as follows:


$$\begin{array}{l} \text{AI }=\;\frac{\text{number of apoptotic cells}}{\text{number of total cells}}\;\times \;100\% \end{array}$$

### Determination of Inflammatory Cytokines *via* Standard Sandwich ELISA

Serum and intestinal tissue samples were collected from the mice, and the expression levels of inflammatory cytokines (IL-4, IL-6, and TNF-α) were detected by using specific ELISA kits. A total of 100 μl of the sample or the standard was added to each well of the ELISA plate. The plates were sealed and incubated at 37°C for 1.5 h. After five cycles of washing, the biotin–antibody diluent and biotinylation antibody working solution were added to the ELISA plates. The plates were sealed and incubated at 37°C for 0.5 h. After five cycles of washing, the substrate solution was added for color development, and the plates were incubated at 37°C for 0.25 h under protection from light. Finally, the termination solution was added and mixed well to terminate the reaction, and the OD450 was measured and recorded immediately.

### Examination of MPO Activity in Intestinal Tissues *via* MPO Colorimetric Assay

MPO activity in intestinal tissues was detected by using a MPO colorimetric activity assay kit. Mouse intestinal tissues were homogenized, mixed thoroughly with buffer, and placed in a water bath at 60°C for 10 min to detect MPO activity. The MPO activity of intestinal tissues was evaluated by measuring the absorbance of the samples at 460 nm (OD). MPO activity was expressed in milliunits per gram weight of wet tissue with the following calculation formula:


$$\begin{array}{l} \text{MPO}\left(\text{U}/\text{g}\right)\;=\;\frac{\text{determination OD value }-\text{ control OD value}}{11.3\;\times \text{ sample volume }\left(\text{g}\right)} \end{array}$$

### Statistical Analysis

Data was expressed as the mean ± standard deviation ($\bar{x}$ ± *s*). Normality test and homogeneity test of variance were performed. SPSS25.0 statistical software was used for data analysis. Data were analyzed with independent-sample *t*-test and one-way analysis of variance followed by Bonferroni. *P* < 0.05 was regarded as statistically significant.

## Results

### Expression Levels of Total BTK and p-BTK in Intestinal Tissue

**Figure 1:**
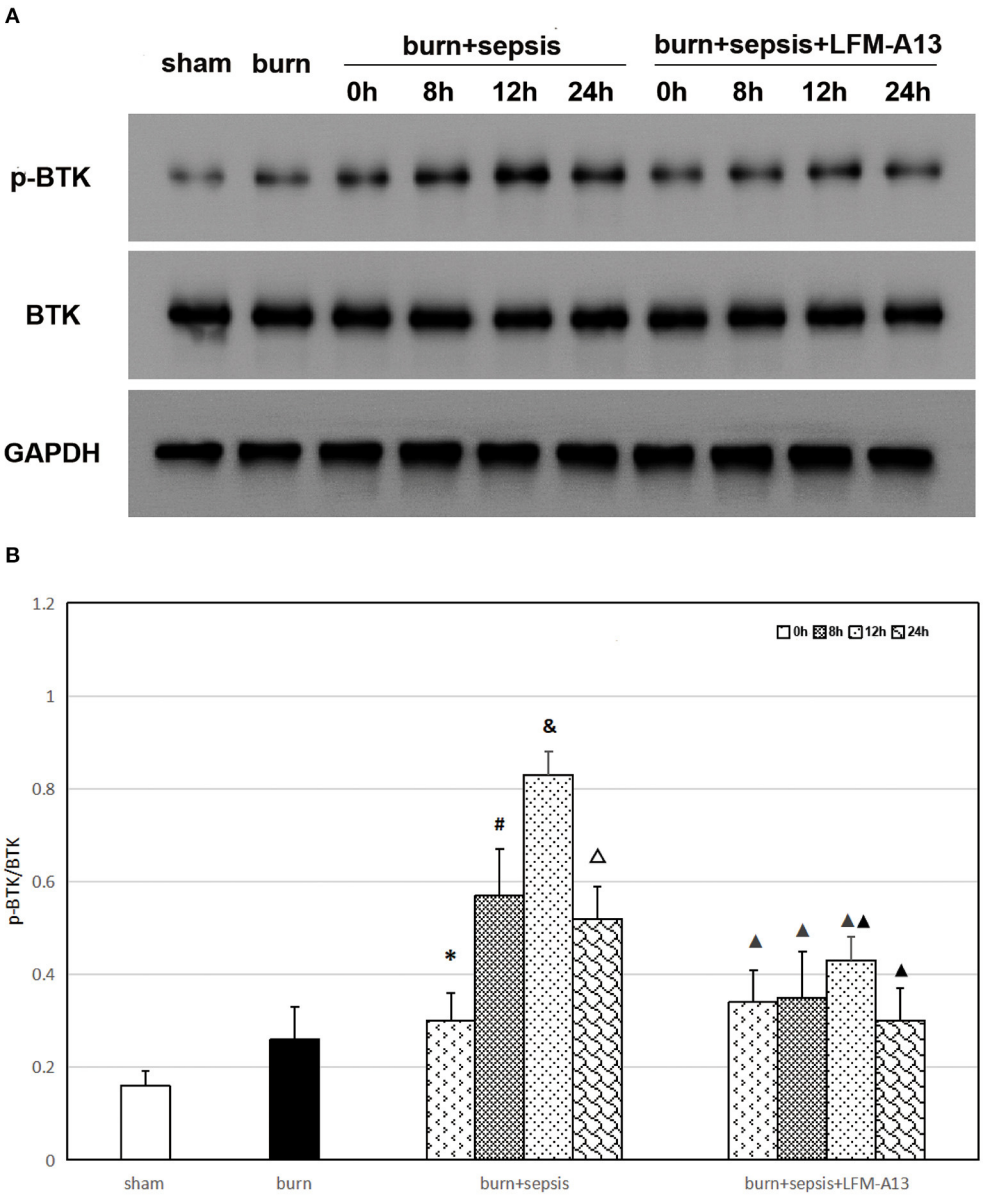
Expression levels of total BTK and p-BTK in intestinal tissue. **(A)** A representative western blot image of total BTK protein and p-BTK in each group. **(B)** The expression level of p-BTK (p-BTK /BTK) in each group at different time points of postburn. ^*^P < 0.05, vs. the sham group and burn group; in the burn + sepsis group, ^#^*P* < 0.05, vs. 0h; ^&^*P* < 0.05, vs. 8h; ^Δ^*P* < 0.05, vs. 12h; ^▴^*P* < 0.05, ^▴▴^*P* < 0.01, vs. the burn + sepsis group at corresponding time points except 0 h of postburn.

As shown in Figure 1, the expression levels of total BTK protein in the intestinal tissues of mice in each group did not significantly differ (*P* > 0.05). Meanwhile, the Western blot analysis results indicated that the p-BTK level in the sham and burn groups were low, whereas that in the burn + sepsis group increased over time, peaked at 12 h, and then decreased at 24 h (*P* < 0.05). The Western blot analysis results of the burn + sepsis + LFM-A13 and burn + sepsis group exhibited similar trends (*P* < 0.05). However, at 8, 12, and 24 h, the expression levels of p-BTK in the burn +sepsis+ LFM-A13 group were significantly decreased relative to those in the burn + sepsis group (*P* < 0.05), indicating that LFM-A13 had a significant inhibitory effect on the activation and expression of BTK in the intestinal tissues of burn sepsis mice.

### Histopathological Analysis and Scores of Intestinal Tissue

**Figure 2:**
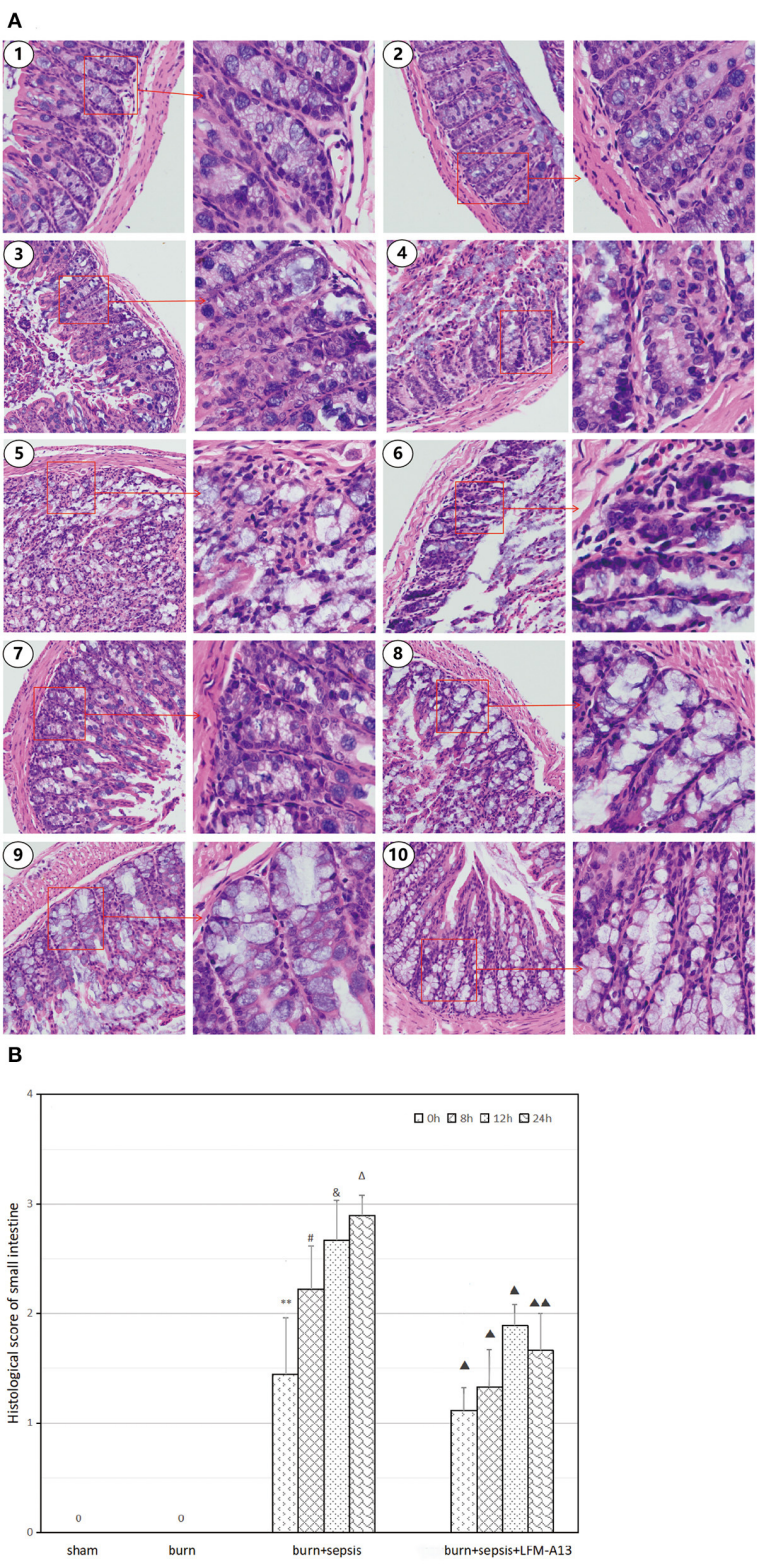
Histopathological analysis and scores of intestinal tissue. **(A)** Histopathological changes in each group. (1) The sham group (×100 and × 400), (2) the burn group (×100 and × 400), (3) the burn+sepsis group at 0 h (×100 and × 400), (4) the burn + sepsis group at 8 h (×100 and × 400), (5) the burn + sepsis group at 12 h (×100 and × 400), (6) the burn + sepsis group at 24 h (×100 and × 400), (7) the burn+sepsis+LFM-A13 group at 0 h (×100 and × 400), (8) the burn+sepsis+LFM-A13 group at 8 h (×100 and × 400), (9) the burn+sepsis+LFM-A13 group at 12 h (×100 and × 400), (10) the burn + sepsis + LFM-A13 group at 24 h (×100 and × 400). **(B)** Histopathology scores in each group at different time points of post-burn. **P < 0.01, vs. the sham group and burn group; in the burn+sepsis group, ^#^*P* > 0.05, vs. 0 h; ^&^*P* > 0.05, vs. 8h; ^Δ^*P* > 0.05, vs. 12h; ^▴^*P* < 0.05, ^▴▴^*P* < 0.01, vs. the burn + sepsis group at corresponding time points except 0 h of post-burn.

As shown in Figure 2, the sham and burn groups received histopathological scores of 0 because they exhibited neatly arranged and intact glands, columnar-lined epithelia, and abundant goblet cells. However, the burn + sepsis group demonstrated obvious histopathological changes, including glandular atrophy, epithelial thinning, and goblet cell reduction or absence that gradually aggravated over time. Meanwhile, the histopathological scores of the burn + sepsis group increased gradually over time (1.44 ± 0.51 vs. 2.22 ± 0.39 vs. 2.77 ± 0.36 vs. 2.89 ± 0.19 vs., *P* > 0.05). Compared with those or the burn+sepsis group at the corresponding time points, the degree of intestinal histopathological changes and pathological scores or the burn+sepsis+LFM-A13 group were significantly lower (1.11 ± 0.19 vs. 1.33 ± 0.33 vs. 1.89 ± 0.19 vs. 1.66 ± 0.34, *P* < 0.05) but were not significantly different at 0 h (*P* > 0.05).

### Apoptosis and AI of Intestinal Epithelial Cells

**Figure 3:**
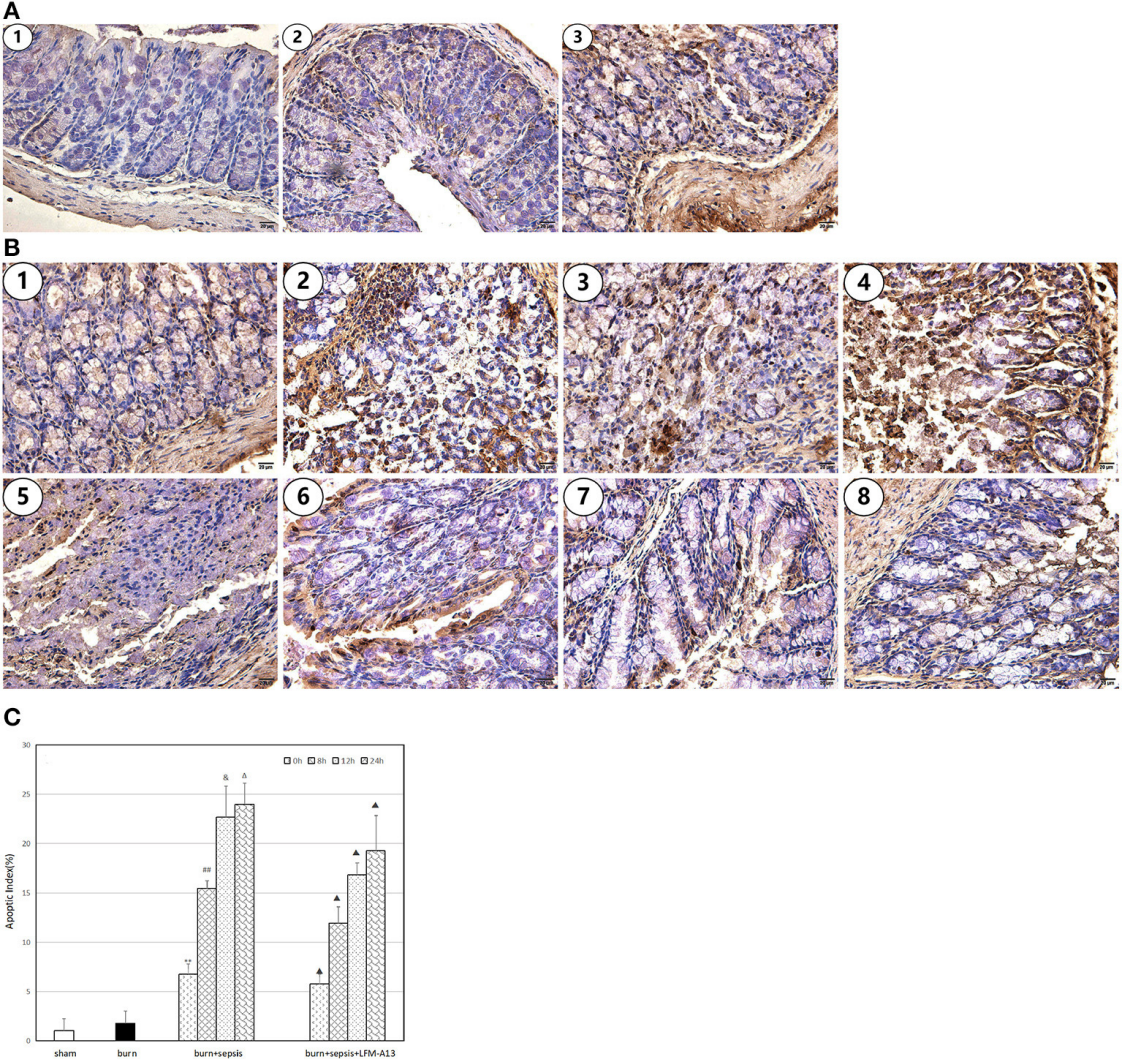
Apoptosis and AI of intestinal epithelial cells. **(A)** Cell apoptosis at 0 h postburn in each group. (1) The sham group, (2) the burn group, (3) the burn + sepsis group. **(B)** Cell apoptosis at 4 different time points of postburn in the burn + sepsis group and burn + sepsis + LFM-A13 group. (1–4): The burn + sepsis group at 0; 8; 12; 24 h, (5–8): the burn + sepsis + LFM-A13 group at 0 h; 8; 12; 24 h. **(C)** AI in each group at different time points of postburn. ^**^P < 0.01, vs. the sham group and burn group; in the burn + sepsis group, ^##^*P* < 0.01, vs. 0 h; ^&^*P* < 0.05, vs. 8 h; ^Δ^*P* > 0.05, *vs* 12 h; ^▴^*P* < 0.05 vs. the burn + sepsis group at corresponding time points except 0 h of post-burn.

Cells with brown or dark brown granules in their nuclei were considered to be apoptotic cells (TUNEL-positive cells). As shown in Figure 3, in the sham group, almost no apoptotic cells were observed in the sections of intestinal tissues, and the AI was 1.06 ± 1.16%. In the burn group, a few apoptotic cells were observed, and the corresponding AI was 1.78 ± 1.25%. However, the apoptotic cells in the burn + sepsis group increased sharply and increased over time (*P* < 0.05). The AIs at 0, 8, and 12 h were 6.77 ± 1.21%, 15.47 ± 0.78%, and 22.71 ± 3.13%, respectively. The corresponding AI of apoptotic cells at 24 h was 23.99 ± 2.14%, and no significant difference was found between the AI at 12 h and that at 24 h. Similarly, compared with that of the burn + sepsis group, the AI of intestinal epithelial cells in the burn + sepsis + LFM-A13 group were significantly lower at the corresponding time points (*P* < 0.05) but did not significantly differ at 0 h (*P* > 0.05).

### Levels of IL-4, IL-6, and TNF-α in Serum and Intestinal Tissue

**Figure 4:**
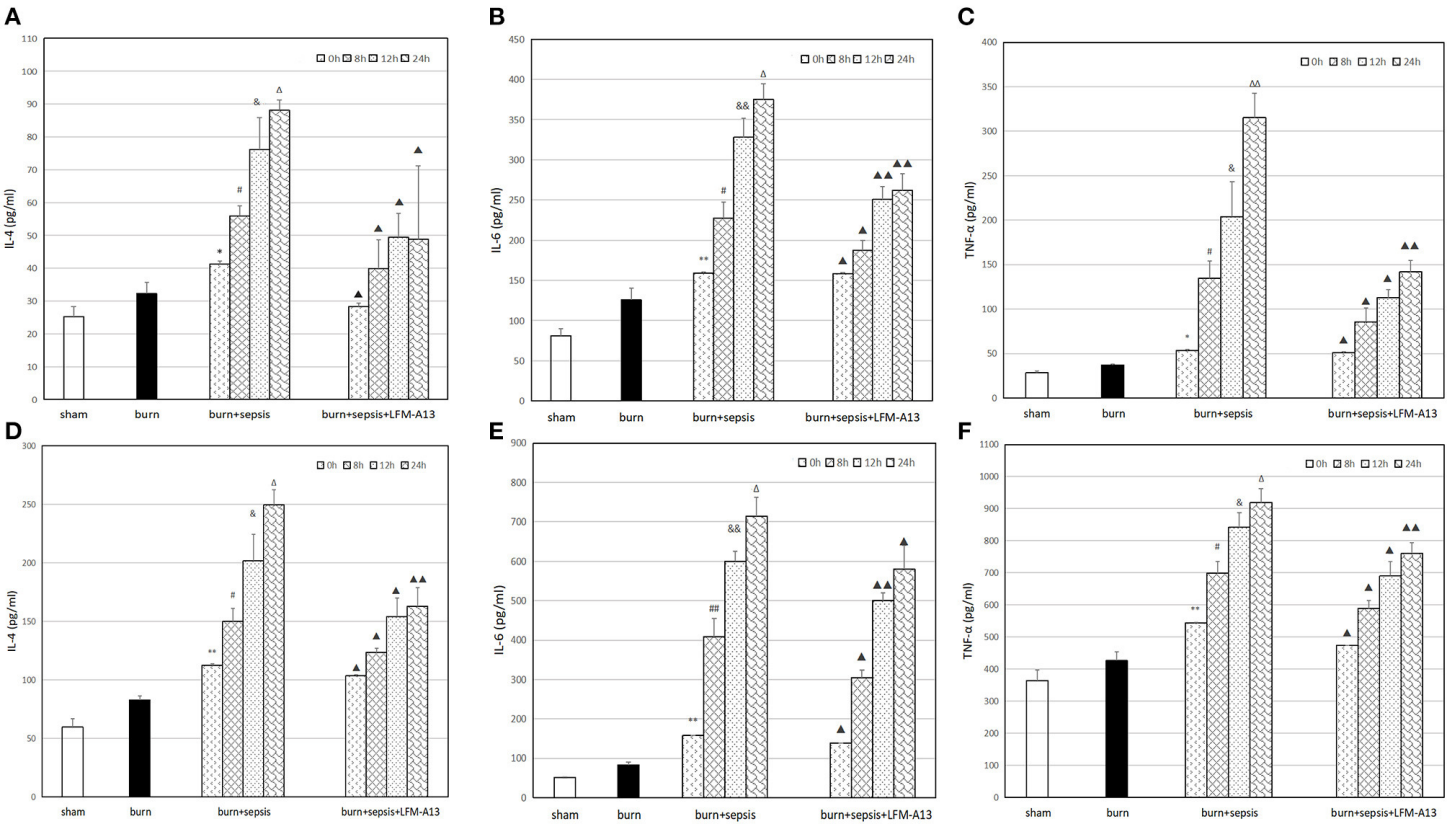
Levels of IL-4, IL-6, and TNF-α in serum and intestinal tissue. **(A–C)**: Levels of IL-4 **(A)**, IL-6 **(B)**, and TNF-α **(C)** levels in serum at different time points in each group. **P < 0.01 **(A,B)**, *P < 0.05 **(C)**, vs. the sham group and burn group; in the burn+sepsis group, *P* < 0.05, ^#^*P* < 0.05 **(A)**,^#^*P* < 0.05 **(B,C)** vs. 0 h, ^&^*P* < 0.05 **(A)**, ^&&^*P* < 0.01 **(B)**, ^&^*P* > 0.05 **(C)** vs. 8 h, ^Δ^*P* > 0.05 **(A,B)**, ^ΔΔ^*P* < 0.01 **(C)** vs. 12 h; ^▴^*P* < 0.05, ^▴▴^*P* < 0.01 vs. the burn+sepsis group at corresponding time points except 0 h postburn. **(D–F)**: Levels of IL-4 **(D)**, IL-6 **(E)** and TNF-α **(F)** levels in intestinal tissue at different time points in each group. **P < 0.01, vs. the sham group and burn group; in the burn+sepsis group, ^#^*P* > 0.05 **(D)**, ^##^*P* < 0.01 **(E)**, ^#^*P* < 0.05 **(F)** vs. 0 h, ^&^*P* < 0.05 **(D,F)**, ^&&^*P* < 0.01 **(E)**, vs. 8 h, ^Δ^*P* < 0.05 **(D,E)**, ^Δ^*P* > 0.05 **(F)**, vs. 12 h; ^▴^*P* < 0.05, ^▴▴^*P* < 0.01, vs. the burn + sepsis group at corresponding time points except 0 h post-burn.

The levels of three kinds of inflammatory cytokines, namely, IL-4, IL-6, and TNF-α, in serum were determined through ELISA. The expression levels of the inflammatory cytokines in each group showed similar trends. As shown in Figures 4A–C, the sham group had the lowest levels of inflammatory cytokines, i.e., IL-4 levels of 25.29 ± 3.07 pg/ml, IL-6 levels of 81.06 ± 8.60 pg/ml, and TNF-α levels of 28.37 ± 1.99 pg/ml. In the burn group, the levels of inflammatory cytokines increased, and the level of IL-4 was 32.27 ± 3.48 pg/ml, that of IL-6 was 125.76 ± 14.29 pg/ml, and that of TNF-α was 37.03 ± 1.40 pg/ml. The levels of the three kinds of inflammatory cytokines in the serum of the burn + sepsis group significantly increased and increased over time (*P* < 0.05). Specifically, IL-4 levels increased from 41.24 ± 4.03 pg/ml (0 h) to 88.19 ± 3.04 pg/ml (24 h); IL-6 levels increased from 159.06 ± 12.84 pg/ml (0 h) to 374.98 ± 19.48 pg/ml (24 h); and TNF-α levels increased from 53.66 ± 16.43 pg/ml (0 h) to 315.49 ± 27.10 pg/ml (24 h). By contrast, the levels of the three inflammatory cytokines, except for those at 0 h (*P* > 0.05), in the burn+sepsis+LFM-A13 group were significantly lower than those in the burn + sepsis group (*P* < 0.05). As shown in Figures 4D–F, the variation trends of the expression levels in each group were consistent with those of the expression levels in serum. Specifically, the expression levels of the inflammatory cytokines in the sham group were the lowest and increased in the burn group. In the burn + sepsis group, the levels of the three inflammatory cytokines in intestinal tissues were also significantly increased and increased over time (*P* < 0.05), whereas those in the burn + sepsis + LFM-A13 group were significantly lower than those in the burn + sepsis group (*P* < 0.05).

### Activity of MPO in Intestinal Tissue

**Figure 5:**
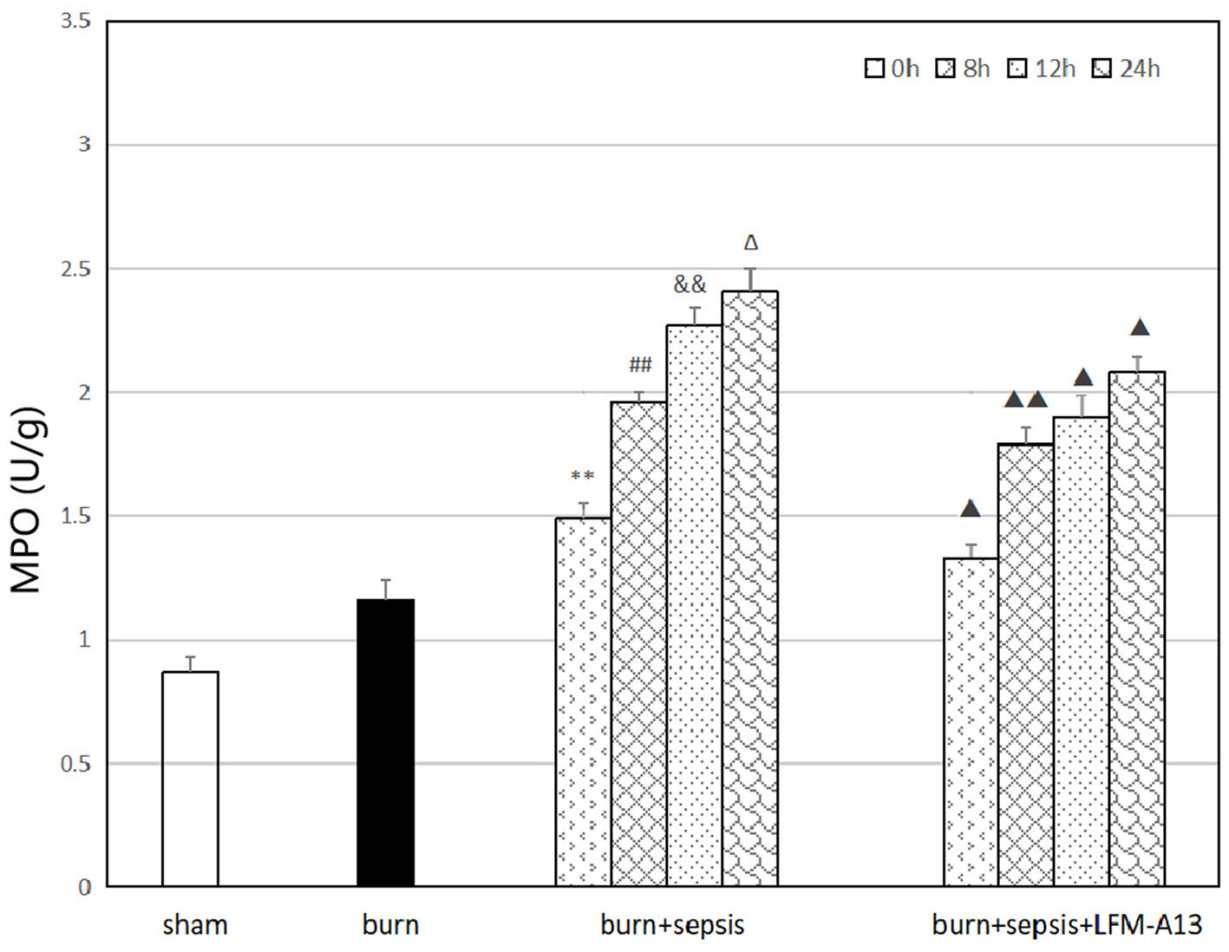
Activity of MPO in intestinal tissue at different time points in each group. **P < 0.01, vs. the sham group and burn group; in the burn + sepsis group, ^##^*P* < 0.01, vs. 0 h, ^&&^*P* < 0.01, vs. 8 h, ^Δ^*P* > 0.05, vs. 12 h; ^▴^*P* < 0.05, ^▴▴^*P* < 0.01, vs. the burn + sepsis group at corresponding time points.

MPO is a functional and activation marker of neutrophils (PMNs), and PMN infiltration in intestinal tissues can be evaluated by assessing MPO activity in intestinal tissues. As shown in Figure 5, the activity of MPO in the burn group was slightly increased compared with that in the sham group, and that in the burn+sepsis group was significantly higher than that in the other two groups and gradually increased over time. It was 1.49 ± 0.05, 1.96 ± 0.04, and 2.27 ± 0.07 U/g at 0, 8, and 12 h, respectively (*P* < 0.05). No significant difference was found between the activity of MPO at 24 h and that at 12 h (*P* > 0.05). Compared with that in the burn+sepsis group, the activity of MPO in the burn + sepsis + LFM-A13 group was significantly lower (*P* < 0.05) but did not significantly differ at 0 h (*P* > 0.05).

## Discussion

Sepsis is a systemic disease that is caused by the dysregulated immune response to infection. It can lead to multisystem organ failure. It involves complex and dynamic processes, including immunity and inflammation, and other aspects with physiological, pathological, and biochemical abnormalities. Burn sepsis, as a type of sepsis, is likely to cause local or systemic inflammatory response syndrome and MODS. In this study, we explored the activation of BTK in the intestinal tissue of burn sepsis mice. Then, we used LFM-A13 to intervene in mice to explore the roles and molecular mechanisms of BTK in burn-sepsis-induced intestinal injury.

Previous studies have shown that TKs are involved in a variety of inflammatory responses in the body and have an important role in autoimmune diseases and tumors (23). As a member of TKs, BTK is involved in the growth and function of B cells and is a key kinase in the B-cell antigen receptor signaling pathway. In this experiment, Western blot analysis was performed to detect the activation of the BTK protein quantitatively. The expression level of total BTK protein in the intestinal tissue of mice in each group clearly had no significant change. The p-BTK protein was expressed at low levels in intestinal tissues of mice in the sham and burn groups. However, the corresponding results of the burn + sepsis group showed that the expression level of p-BTK increased over time, peaked at 12 h, then decreased at 24 h. Similarly, the histopathological results also showed no obvious pathological changes in the sham and burn groups, whereas the degree of histopathological damage in the burn + sepsis group gradually intensified over time, peaked at 12 h, and then decreased at 24 h. Therefore, these results demonstrated that BTK activation was closely associated with the degree of intestinal injury and played a destructive role in intestinal injury.

TK inhibitors can be used to inhibit the abnormal pathways of cell signal transduction and participate in cell growth and proliferation. LFM-A13 is an inhibitor with relatively good targeting capability; it can inhibit some members of the Tec family, such as BTK, by inhibiting the activation of BTK in cells and thus inhibits excessive inflammatory responses in the body (24). In this study, one of the groups of mice received LFM-A13 intervention immediately after burn sepsis modeling, and their BTK expression levels were observed at 0, 8, 12, and 24 h after injury. The results showed that the expression levels of the total BTK protein in the intestinal tissue of mice in each group did not significantly differ. Compared with those in the burn+sepsis group at the corresponding time points (8, 12, and 24 h), the expression levels of the p-BTK protein in the burn + sepsis + LFM-A13 group had significantly decreased. H&E staining also showed that at corresponding time points (8, 12, and 24 h), the degree of histopathological changes in the burn + sepsis + LFM-A13 group was significantly reduced compared with that in the burn + sepsis group, and the pathological scores were significantly reduced. These results further demonstrated that in the burn sepsis-induced intestinal injury of mice, the activation of BTK could be inhibited significantly by LFM-A13. Our previous study also showed that LFM-A13 inhibited BTK activation and inhibited the burn sepsis-induced pyroptosis of intestinal cells (22).

In the present study, the apoptosis of intestinal epithelial cells and AIs were detected by using the TUNEL assay. As can be clearly seen, apoptotic cells were rare in the sections of intestinal tissues from the sham and burn groups. However, the number of apoptotic cells increased sharply in the burn + sepsis group, and the AIs increased accordingly. These results indicated that in burn sepsis mice, the activation of BTK was closely associated with the apoptosis of intestinal epithelial cells. The TUNEL assay was also used to detect the apoptotic cells in the intestinal tissues of mice in the burn + sepsis + LFM-A13 group to illustrate this point. At the corresponding time points, the number of apoptotic cells significantly decreased and the AI decreased accordingly in the burn + sepsis + LFM-A13 group compared with those in the burn + sepsis group. These results also indicated that LFM-A13 could significantly reduce the apoptosis of intestinal epithelial cells in burn sepsis mice by inhibiting the activation and expression of BTK during intestinal injury and thus played a protective role against intestinal injury in burn sepsis mice.

Previous studies have shown that cell apoptosis is mainly induced by the activation of Caspase-3, which acts as the final downstream protein required for cell apoptosis (25). Bcl-2 and Bax, the main antiapoptotic proteins of the Bcl family, could execute apoptotic programs by regulating Caspase-3 during apoptosis (26, 27). This study found that LFM-A13 could significantly reduce the apoptosis of intestinal epithelial cells in burn sepsis mice. Thus, whether LFM-A13 could reduce the apoptosis of cells by inhibiting the activation of Caspase-3, by activating Bcl-2 and Bax, or by doing both remains unclear. The mechanisms of LFM-A13 in inhibiting apoptosis in intestinal tissues warrant further studies.

Previous studies have shown that the main role of the gastrointestinal tract in the body is digestion and absorption and is often regarded as a passive organ during the occurrence and development of sepsis and resistance to invasion by pathogenic microorganisms (28). However, with the deepening of research in recent years, the intestinal metabolism of the body has been found to be abnormal in the early stage of sepsis and that the intestine is vulnerable to damage during sepsis, causing multiple organ dysfunction (29). In a normal organism, the intestinal mucosal barrier could keep endotoxins and bacteria in the gut away from the blood and allow the body to function normally (30). Severe burns cause a wide range of immune responses and extensive cell damage, which is considered a precursor to organ dysfunction. Then, excessive inflammatory reaction would destroy endothelial and intestinal epithelial cells and lead to their apoptosis, resulting in increased intestinal permeability and aggravating intestinal mucosal barrier dysfunction; these effects are followed by endotoxin and bacterial migration and further intestinal flora imbalance (31). As a result, sepsis in patients progressively develops and deteriorates. Among the many known inflammatory mediators, three inflammatory factors (IL-4, IL-6, and TNF-α) play an important role in local or systemic inflammatory response (32). They could release secondary inflammatory factors by stimulating immunoreactive cells, such as monocytes and macrophages, to cause intestinal tissue inflammation. They could also promote the release of proteases and oxygen free radicals from PMNs, causing inflammatory injury (33).

ELISA results showed that the levels of inflammatory factors in the burn sepsis group were significantly higher than those in the other two groups and that IL-4, IL-6, and TNF-α continuously increased over time. Therefore, these results showed that the activation of BTK was closely related to the release of inflammatory factors in the serum and intestinal tissue of burn sepsis mice. Compared with those in the burn+sepsis group at the corresponding time points (8, 12, and 24 h), the levels of inflammatory cytokines in serum and intestinal tissue were significantly decreased in the group treated with LFM-A13. These results indicated that in burn sepsis mice, LFM-A13 could significantly inhibit the expression of inflammatory cytokines by inhibiting the activation and expression of BTK during intestinal injury, thereby reducing the excessive inflammatory response and exerting a protective effect.

MPO, a peroxidase present in myeloid cells, is a specific marker of myeloid cells and a marker of the function and activation of PMNs; changes in its expression level and activity represent the function and activity of PMNs (34). The detection of MPO activity in the intestinal tissues of mice showed that the activity of MPO in the burn+sepsis group was significantly higher than that in the sham and burn groups and gradually increased over time. Therefore, the results suggested that in the intestinal tissues of burn sepsis mice, the activation of BTK was closely associated with the activity of MPO. MPO catalyzes the conversion of H_2_O_2_ and Cl^−^ to form OCl^−^ and free radicals with oxidative capacity; the MPO–H_2_O_2_-Cl^−^ system is then formed to regulate the immune response of the body. Meanwhile, MPO can activate signaling pathways, such as the NF-κB pathway, to promote the development of inflammatory response. At the corresponding time points (8, 12, and 24 h), the activity of MPO in the burn + sepsis + LFM-A13 group was significantly lower than that in the burn + sepsis group. These results indicated that in burn sepsis mice, the BTK-specific inhibitor LFM-A13 could significantly inhibit the expression of MPO in intestinal tissues by inhibiting the activation and expression of BTK during intestinal injury, thus mitigating the oxidative stress response.

## Conclusion

BTK signaling pathway mediated intestinal cell apoptosis; regulated the production of the anti-inflammatory cytokine IL-4 and the proinflammatory cytokines IL-6 and TNF-α; and participated in intestinal oxidative stress. Thus, it played an important regulatory role in the intestinal injury induced by burn sepsis.

## Data Availability Statement

The original contributions presented in the study are included in the article/supplementary materials, further inquiries can be directed to the corresponding author/s.

## Ethics Statement

The animal study was reviewed and approved by Anhui Medical University Ethics Committee of Animal Experiments.

## Author Contributions

X-LC developed the idea, designed the study, and provided financial support for the study. JW performed the experiment, drafted the manuscript, summarized the data, and contributed to data interpretation. XY, J-QN, LQ, and FW were involved in the acquisition of the data. The corresponding author had full access to all the data in the study and was responsible for submission for publication. All authors contributed to the article and approved the submitted version.

## Funding

This work was supported by the National Natural Science Foundation of China (Grant No. 82172204).

## Conflict of Interest

The authors declare that the research was conducted in the absence of any commercial or financial relationships that could be construed as a potential conflict of interest.

## Publisher's Note

All claims expressed in this article are solely those of the authors and do not necessarily represent those of their affiliated organizations, or those of the publisher, the editors and the reviewers. Any product that may be evaluated in this article, or claim that may be made by its manufacturer, is not guaranteed or endorsed by the publisher.
